# Predictors of response to burosumab in adults with X-linked hypophosphatemia: real-world data from an Italian cohort

**DOI:** 10.1007/s40618-025-02596-3

**Published:** 2025-05-05

**Authors:** Gaetano Paride Arcidiacono, Valentina Camozzi, Giovanni Tripepi, Cristina Eller-Vainicher, Giuseppe Vezzoli, Maria Luisa Brandi, Gemma Marcucci, Giuseppe Girasole, Antonio Aversa, Corrado Vitale, Gaetana Cerbone, Maria Michela D’Alessandro, Martina Zaninotto, Maria Fusaro, Marco Onofrio Torres, Michele Cannito, Alberta Cecchinato, Martin Diogo, Mor Peleg Falb, Francesca Guidolin, Marta Zampogna, Mario Plebani, Elena Campello, Paolo Simioni, Stefania Sella, Sandro Giannini

**Affiliations:** 1 Department of Medicine, Clinica Medica 1, University of Padova, Padua, Italy; 2 Endocrinology Unit, University Hospital of Padova, Padua, Italy; 3 Institute of Clinical Physiology (IFC), Clinical Epidemiology of Renal Diseases and Hypertension, National Research Council (CNR), Ospedali Riuniti, Reggio Calabria, Italy; 4 Fondazione IRCCS, Cà Granda Ospedale Maggiore Policlinico, Milan, Italy; 5 Nephrology and Dialysis Unit, IRCCS San Raffaele Scientific Institute, Milan, Italy; 6 Fondazione FIRMO Onlus (Fondazione Italiana Ricerca Sulle Malattie Dell’Osso), Florence, Italy; 7 Department of Experimental and Clinical Biomedical Sciences, University of Florence, Florence, Italy; 8 Rheumatology Department, “La Colletta” Hospital, ASL 3 Genovese, Arenzano, Italy; 9 Department of Experimental and Clinical Medicine, Magna Graecia University of Catanzaro, Catanzaro, Italy; 10 Nephrology and Dialysis Unit, Azienda Ospedaliera Ordine Mauriziano di Torino, Turin, Italy; 11 Division of Medical Genetics, “S.G. Moscati” Hospital, Avellino, Italy; 12 Pediatric Nephrology Unit, Azienda di Rilievo Nazionale ed Alta Specializzazione (ARNAS) Civico, Di Cristina, Benfratelli, Palermo, Italy; 13 QI.LAB.MED, Spin-off dell’Università di Padova, Padua, Italy; 14 Institute of Clinical Physiology (IFC), National Research Council (CNR), Pisa, Italy; 15 Department of Medicine, University of Padova, Padua, Italy

**Keywords:** Burosumab, X-linked hypophosphatemia, Serum phosphate, FGF23, Patient-reported outcomes, Real-life

## Abstract

**Purpose:**

X-linked hypophosphatemia (XLH) is a genetic disorder characterized by elevated FGF23 levels, leading to phosphate wasting and hypophosphatemia, causing skeletal and extraskeletal abnormalities. Burosumab, an antibody targeting FGF23, improves hypophosphatemia and clinical outcomes. This study evaluated the real-world efficacy of burosumab and identify predictors of treatment response.

**Methods:**

Twenty-seven adult XLH patients (mean age 42 years; 48% female) from an Italian multicenter cohort were treated with burosumab for up to 24 weeks. Laboratory tests were evaluated at midpoints and endpoints (14 and 28 days) of the dosing interval. In a subset of patients (N = 11) followed for 48 weeks, laboratory tests and patient-reported outcomes were also assessed.

**Results:**

After initiating burosumab, median serum phosphate levels increased from 1.5 mg/dL (IQR 1.3–1.8) to 2.0 mg/dL (IQR 1.7–2.4) (p < 0.05), remaining higher than baseline at the midpoints of the dosing interval for up to 24 weeks. Higher baseline phosphate predicted higher midpoint levels (p < 0.05), whereas higher baseline PTH (p < 0.05) and FGF23 (p < 0.001) were associated with lower phosphate levels at midpoints. In patients (N = 11) followed for 48 weeks, significant improvements in patient-reported outcomes in all patients were observed. Both WOMAC Pain (r = 0.94, p = 0.02) and BPI Worst Pain (r = 0.98, p < 0.001) were positively correlated with increased phosphate at week 48.

**Conclusion:**

Burosumab effectively increased serum phosphate levels and improved clinical outcomes in a real-world setting, particularly in patients with more substantial increases in serum phosphate levels. Baseline serum phosphate, PTH, and FGF23 levels predicted response, helping tailor treatment strategies and improve long-term patient management.

**Supplementary Information:**

The online version contains supplementary material available at 10.1007/s40618-025-02596-3.

## Introduction

X-linked Hypophosphatemia (XLH) is a rare genetic disease caused by mutations in the PHEX gene (Xp22.11) [1, 2]. These mutations impair the function of the phosphate-regulating endopeptidase homolog, X-linked (PHEX), leading to increased circulating levels of bone-derived phosphaturic hormone Fibroblast Growth Factor 23 (FGF23) [3–5]. FGF23 is a key regulator of phosphate homeostasis, and elevated levels of FGF23 are associated with urinary phosphate wasting and chronic hypophosphatemia [6]. This disruption of phosphate metabolism contributes to the development of osteomalacia and rickets [7], which are the main features of XLH, along with bone pain, muscle weakness, and dental anomalies [8, 9].

Conventional treatment for XLH involves the administration of phosphate salts and active vitamin D analogs (such as calcitriol or alfacalcidol) to counteract the consequences of hypophosphatemia. However, phosphate and calcitriol therapy is often unable to achieve satisfactory results (even with adherence to treatment) in terms of height achievement, improvement in bone deformities and pain control [10, 11]. Although effective, especially during periods of growth, this treatment is linked to several common side effects, such as hypercalciuria, nephrocalcinosis, secondary or tertiary hyperparathyroidism, and gastrointestinal issues related to phosphate supplementation (such as nausea, diarrhea, and abdominal discomfort) [4]. These side effects can affect adherence to treatment, potentially compromising its effectiveness.

Recently, burosumab, a human monoclonal antibody that inactivates FGF23, has become available for the treatment of XLH [10]. Unlike conventional therapy with calcitriol and phosphate salts, burosumab directly targets and counteracts the activities of FGF23, addressing the pathogenetic mechanism of XLH more specifically. Burosumab has proven to be effective in children and adult patients in improving serum phosphate levels, other biochemical markers of the disease (e.g., tubular maximum phosphate reabsorption per glomerular filtration rate [TmP/GFR], 1,25-(OH)_2_-vitamin D, alkaline phosphatase), as well as clinical outcomes [12–14, 14, 15].

Burosumab became available for adults in Italy through an Early Access Program (EAP) in 2019 and was subsequently approved by the National Regulatory Authority (AIFA, Italian Medicines Agency) in 2023 [16]. The criteria for drug access under EAP and subsequently for reimbursement in Italy are likely more stringent than those of the pivotal trial [17]. EAP criteria included the presence of active fractures or pseudofractures, moderate to severe pain impacting quality of life (QoL), and/or the need for orthopedic surgery, dental surgery involving bone, or spinal surgery within a predefined period before and after the intervention. AIFA criteria included the presence of ongoing hyperphosphaturic hypophosphatemia, active fracture/pseudofractures, and/or uncontrolled pain, assessed using the BPI and WOMAC scores. Moreover, both for EAP and AIFA, disease manifestations had to be inadequately controlled by conventional therapy with phosphate and active vitamin D analogs alone. In this study, we aimed to analyze the experience of using burosumab in Italian adult patients with XLH, assessing the relationship between clinical and biochemical responses to the drug in this real-world setting.

## Materials and methods

### Patients

We enrolled adult patients with XLH evaluated in the outpatient clinic of nine University Hospitals in Italy (University Hospital of Padova, “Fondazione Ca’ Granda IRCCS Ospedale Maggiore Policlinico” of Milano, “San Raffaele” Hospital and “Università Vita Salute San Raffaele” of Milano, “Ordine Mauriziano” Hospital of Torino, University-Hospital “Careggi” of Firenze, “Azienda Sanitaria Locale 3—Ospedale “La Colletta” of Arenzano, University Hospital “Mater Domini” of Catanzaro, “San Giuseppe Moscati” Hospital of Avellino, and “Civico Di Cristina e Benfratelli” Hospital of Palermo from 2019 to 2023, when burosumab treatment became available in Italy, through an EAP in a first phase and then by using the same drug available on the pharmaceutical market.

The diagnosis of XLH was based on the presence of the following: (1) chronic hypophosphatemia (i.e., fasting serum phosphate levels persistently below 2.5 mg/dL before initiating any phosphate or calcitriol supplementation); (2) renal phosphate wasting (i.e., TmP/GFR below the age and sex-adjusted reference range [18]); (3) high serum FGF23 levels; (4) presence of PHEX mutations and/or family history of XLH; (5) clinical features suggestive of XLH (e.g., short stature, lower limb deformities, pseudofractures, recurrent dental abnormalities) [19].

Inclusion criteria for the study were: (1) age ≥ 18 years; (2) treatment with burosumab for at least 24 weeks. Exclusion criteria were: chronic kidney disease (CKD) stages 3–5 (estimated glomerular filtration rate [eGFR] < 60 mL/min/1.73 m^2^), acute or chronic liver failure, presence of malignant neoplasm, previous or current use of anti-osteoporosis drugs and medications able to modulate parathyroid hormone (PTH) (e.g., cinacalcet), use of diuretics, chronic use of systemic corticosteroids, pregnancy, or breastfeeding. None of the enrolled patients were members of the same family (i.e. all participants were unrelated).

### Data collection

Medical records were reviewed retrospectively, and demographic, clinical, and radiological data were collected for all patients, where available. These included age, sex, height, weight, body mass index (BMI), past medical history, clinical features of XLH (e.g., presence of PHEX mutations, family history, history of skeletal deformities and/or previous orthopedic surgery, history of fragility fractures and enthesopathies, history of dental abnormalities and nephrolithiasis), and ongoing medical treatments.

### Biochemical analysis

Patients underwent laboratory tests at baseline, as well as at midpoints and endpoints of dosing intervals. Baseline fasting blood and urine samples, including serum and urinary calcium and phosphate, serum and urinary creatinine, 25-OH-vitamin D, PTH and intact FGF23 (iFGF23) were collected and analyzed at single center level. Given the different reference ranges for PTH and iFGF23 used across the nine centers, these two variables were expressed as standard deviations from the mean of the respective reference population using the z-log transformation method for the multicenter analysis [20]. In addition, serum phosphate levels were measured at both midpoints and endpoints of the dosing intervals (see below).

In the subgroup analysis of patients evaluated at the University Hospital of Padova, blood and urine samples were collected for biochemical analysis at the Laboratory Medicine Unit of the University of Padova, using methods whose quality performance is monitored according to the ISO 15189 standard. Lithium-heparin plasma and 24-h urine samples were collected for calcium and phosphate measurements using colorimetric methods and for creatinine enzymatic assay (with calibration traceable to the reference procedure). Serum 25-OH-vitamin D, 1,25-(OH)_2_-vitamin D, PTH (third-generation assay), and intact FGF23 (plasma K2-EDTA) were measured using automated immunochemiluminescent methods (Liaison XL, DiaSorin, Saluggia, Italy). Bone alkaline phosphatase (bALP) was measured with an immunochemiluminescence method (IDS-iSYS, Pantec, Turin, Italy).

eGFR was calculated with the 2021 Chronic Kidney Disease Epidemiology Collaboration [CKD-EPI] Creatinine equation [21]. TmP/GFR was calculated on fasting blood and morning urine sample as previously described [22].

### Patients’ assessments during burosumab treatment

In the entire cohort of patients included in the study, burosumab was administered via in-hospital subcutaneous injection at the recommended dose of 1.0 mg/kg of body weight, rounded to the nearest 10 mg up to a maximum dose of 90 mg, every 4 weeks for at least 24 weeks. Burosumab was initiated seven days after the suspension of conventional therapy (phosphate supplements and/or active vitamin D analogs). Timepoints corresponding to 2 and 4 weeks after burosumab administration were defined as midpoints and endpoints, respectively. In the subgroup of patients evaluated at the University Hospital of Padova, the treatment duration was 48 weeks. These patients also completed the following questionnaires at baseline and after 48 weeks of burosumab treatment [23]. The Brief Pain Inventory Short Form (BPI-SF) was used to assess the severity of pain and the extent to which pain interferes with daily activities [23]. Western Ontario and McMaster Universities Osteoarthritis Index (WOMAC) to assess pain, stiffness, and physical functioning [23]. The Health Assessment Questionnaire (HAQ) and the Routine Assessment of Patient Index Data 3 (RAPID3) were used to evaluate health-related QoL [24].

### Statistical analysis

Normally distributed continuous variables are expressed as mean ± standard deviation (SD). Non-normally distributed continuous variables are expressed as median and interquartile range (IQR). The T-test for independent observations was used to compare normally distributed continuous variables, and the Mann–Whitney test was used for non-normally distributed continuous variables between two groups. For paired comparisons, the paired T-test was used for normally distributed continuous variables, and the Wilcoxon signed-rank test was used for non-normally distributed continuous variables. The chi-square test or Fisher’s exact test was used to compare categorical variables. Pearson or Spearman correlation coefficients were used to assess the correlation between variables, depending on the data distribution. Univariable and multivariable linear regression analysis was used to examine the relationships among variables; results were expressed as regression coefficients (B) and p-values.

The calculation of the receiver operating characteristic (ROC) curves and related areas under the curve (AUC) were used to evaluate the diagnostic performance of the variables; the optimal cut-off value was determined by calculating the Youden index. The results were considered significant when the p-value was less than 0.05. Statistical analysis was performed using the software Statistical Package for the Social Sciences (SPSS Statistics for Windows, Version 27.0. Armonk, NY: IBM Corp).

## Results

### Patients’ characteristics

A total of 27 adult patients with XLH were enrolled (Table 1). Patients had a mean age of 42 years (range 18–62), and 48% were female. In 23 patients, there was a confirmed PHEX gene mutation, while genetic analysis was not available for 4 patients, who instead had a positive family history. The clinical manifestations associated with XLH in patients are also reported in Table 1. Serum phosphate levels were significantly below the lower limit of normal (median 1.5 mg/dL, IQR 1.3–1.8), as was TmP/GFR (median 1.3 mg/dL, IQR 0.9–1.5). Serum and urinary calcium, serum creatinine, and eGFR were within the normal range in all patients. All patients were receiving phosphate and/or calcitriol therapy prior to initiating burosumab treatment. Common side effects associated with conventional therapy include secondary hyperparathyroidism, nephrolithiasis, and nephrocalcinosis. In our cohort, no patient had a diagnosis of nephrocalcinosis, while nephrolithiasis was present in 33% (9/27) of patients (Table 1). After withdrawal from any phosphate or active vitamin D analogue therapies, all patients began treatment with burosumab at a median dose of 60 (IQR 53–78) mg, corresponding to 1.00 (IQR 0.96–1.03) mg per kg, every 4 weeks. No dose adjustments occurred during the observation period, and no significant adverse events were reported (e.g., hypersensitivity reactions, hyperphosphatemia, kidney stones).

**Table 1:** Patients’ baseline characteristics

Variables	XLH (n = 27)
*General*	
Age (years)	42 ± 13
Female sex, n (%)	13 (48)
Weight (Kg)	60 (55–78)
Height (cm)	150 (144–155)
BMI (kg/m^2^)	27 (25–32)
*XLH-related clinical data*	
PHEX mutation, n (%)	23 (85)
Family history of rickets, n (%)	8 (30)
Lower limb deformities and/or previous orthopedic surgery, n (%)	25 (93)
Fragility fractures/pseudofractures, n (%)	19 (70)
Enthesopathies, n (%)	16 (59)
Nephrolithiasis, n (%)	9 (33)
Dental abnormalities, n (%)	16 (59)
*Laboratory tests*	
Serum phosphate (mg/dL)	1.5 (1.3–1.8)
TmP/GFR (mg/dL)	1.3 (0.9–1.5)
Serum calcium (mg/dL)	9.4 (9.0–9.6)
Urinary calcium (mg/24 h)	119 (82–148)
25-OH-vitamin D (nmol/L)	64 (49–89)
Serum creatinine (mg/dL)	0.65 (0.57–0.74)
eGFR (ml/min/1.73 m^2^)	115 (109–126)

Data presented as mean ± SD or median and interquartile range

BMI = body mass index, eGFR = estimated glomerular filtration rate, PHEX = phosphate regulating endopeptidase X-linked, TmP/GFR = tubular maximum phosphate reabsorption per glomerular filtration rate, XLH = X-linked hypophosphatemia

### Whole group analysis

#### Trend of serum phosphate over 24 weeks of burosumab treatment

**Fig. 1:**
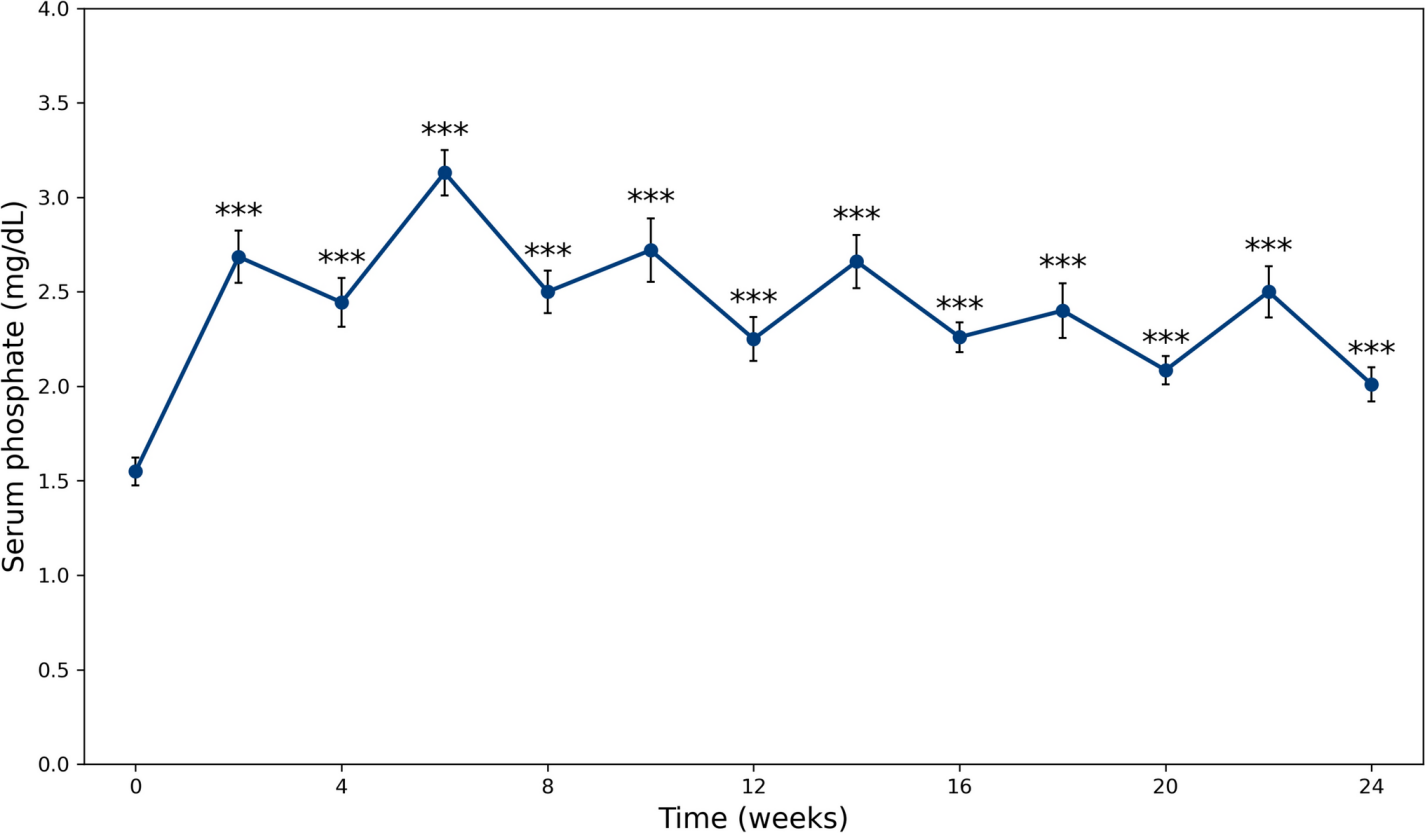
Trends in serum phosphate up to week 24, comparing each timepoint with baseline. * represents a p-value of < 0.05, ** = p < 0.01 and *** = p < 0.001

After the first administration of burosumab, serum phosphate levels increased from a baseline of 1.5 mg/dL to a peak of 2.7 mg/dL (IQR 2.4–3.3) at week 2, and then decreased to 2.4 mg/dL (IQR 2.2–2.8) by week 4. By the end of the observation period, serum phosphate at week 24 was 2.0 mg/dL (IQR 1.7–2.4). Serum phosphate levels at all considered timepoints during burosumab treatment were significantly higher than baseline (Fig. 1, p < 0.05 for all comparisons).

As in the pivotal trial by Insogna et al. [17], we decided to calculate, for each patient, the mean of serum phosphate levels at midpoints over the 24 weeks of treatment as a laboratory efficacy outcome. The median value of this variable for the entire cohort was 2.7 mg/dL (IQR 2.3–3.3), with a median percentage increase of 86% (IQR 85–132%) compared to baseline. We also observed that 17 patients (63%) achieved a mean serum phosphate at midpoints of 2.5 mg/dL or higher. Consequently, we divided the patients into two groups based on their mean serum phosphate at midpoints: lower (< 2.5 mg/dL) and higher (≥ 2.5 mg/dL). Supplementary Fig. 1 shows the different trends of serum phosphate in the two groups. We observed that serum phosphate levels were consistently more elevated in the latter group at all considered timepoints (p < 0.05 for all comparisons). Compared to serum baseline phosphate, in the former group we observed a median percentage increase of 51% (IQR 42–75), while in the latter group, the increase was 95% (IQR 73–135).

#### Factors associated with serum phosphate levels at midpoints during 24 weeks of burosumab treatment

We analyzed baseline factors associated with mean serum phosphate levels at midpoints using a linear regression model. As shown in Table 2, baseline serum phosphate levels were positively associated with mean serum phosphate at midpoints, while TmP/GFR exhibited a similar but non-significant trend. As described in the Methods section, PTH and FGF23 values were ZLOG-transformed using the respective laboratory reference ranges. Both baseline PTH and FGF23 levels were negatively associated with mean serum phosphate at midpoints. A multivariable regression analysis was subsequently performed to further explore the factors associated with mean serum phosphate at midpoints. Due to the sample size and the established influence of serum PTH and FGF23 on serum phosphate, only these two variables were included in the model. ZLOG PTH was not significantly associated with serum phosphate (b = − 0.138, p = 0.298), whereas ZLOG FGF23 showed a significant negative association (b = − 0.165, p = 0.020). The adjusted R-squared value for the model was 0.528, indicating that approximately 52.8% of the variance in mean serum phosphate levels at midpoints was explained by the included predictors.

**Table 2:** Univariable linear regression analysis with mean serum phosphate levels at midpoints as the dependent variable

Predictor variable (units of increase)	Univariable linear regression		
	Coefficient (B)	Standard error (SE)	p-value
*General*			
Age (10 years)	0.032	0.093	0.735
Sex (male vs. female)	− 0.243	0.246	0.334
Height (10 cm)	0.214	0.105	0.053
Weight (10 kg)	− 0.041	0.088	0.645
BMI (1 kg/m^2^)	− 0.041	0.020	0.058
4-week dose of burosumab (10 mg)	− 0.038	0.088	0.673
*Baseline laboratory tests*			
Serum phosphate (1 mg/dL)	0.697	0.305	**0.032**
TmP/GFR (1 mg/dL)	0.694	0.333	0.051
Serum calcium (1 mg/dL)	− 0.194	0.238	0.424
Urinary calcium (10 mg/24 h)	0.003	0.023	0.905
Serum creatinine (1 mg/dL)	− 0.207	0.753	0.786
eGFR (10 ml/min/1.73 m^2^)	− 0.034	0.072	0.643
25-OH-vitamin D (10 nmol/L)	0.035	0.039	0.372
ZLOG iFGF23 (1 SD)	− 0.206	0.050	**0.001**
ZLOG PTH (1 SD)	− 0.175	0.085	**0.048**

Statistically significant p-values (< 0.05) are indicated in bold

BMI = body mass index, eGFR = estimated glomerular filtration rate, iFGF23 = intact fibroblast growth factor 23, PTH = parathyroid hormone, TmP/GFR = tubular maximum phosphate reabsorption per glomerular filtration rate, SD = standard deviation

### Subgroup analysis

We then conducted a subgroup analysis using data only from the 11 patients evaluated at the University Hospital of Padova, who were followed for 48 weeks. No significant differences were observed between these patients and patients enrolled in the other Centers in demographic, anthropometric, clinical and laboratory parameters, except for a slight difference in baseline serum phosphate and TmP/GFR values was observed (Table 3).

**Table 3:** Baseline characteristics of patients by cohort

Variables	Padova patients (n = 11)	Other Centers’ patients (n = 16)	p-value
*General*			
Age (years)	41 (34–55)	42 (26–56)	0.77
Female sex, n (%)	7 (63)	6 (38)	0.18
Weight (Kg)	57 (52–61)	67 (57–82)	0.05
Height (cm)	149 (146–154)	153 (142–159)	0.40
BMI (kg/m^2^)	26 (24–27)	29 (26–33)	0.19
*XLH-related clinical data*			
Family history of rickets, n (%)	4 (36)	4 (25)	0.53
Lower limb deformities and/or previous orthopedic surgery, n (%)	11 (100)	14 (88)	0.22
Fragility fractures/pseudofractures, n (%)	8 (73)	11 (69)	0.82
Enthesopathies, n (%)	5 (45)	11 (69)	0.34
Nephrolithiasis, n (%)	4 (36)	5 (31)	0.59
Dental abnormalities, n (%)	8 (73)	8 (50)	0.17
*Laboratory tests*			
Serum phosphate (mg/dL)	1.3 (1.0–1.5)	1.7 (1.5–2.1)	** < 0.01**
TmP/GFR (mg/dL)	0.9 (0.8–1.1)	1.4 (1.3–1.6)	**0.02**
Serum calcium (mg/dL)	9.2 (8.8–9.7)	9.4 (9.2–9.5)	0.40
25-OH-vitamin D (nmol/L)	64 (53–87)	63 (40–98)	0.90

Data presented as mean ± SD or median and interquartile range. Statistically significant p-values (< 0.05) are indicated in bold

bALP = bone alkaline phosphatase, BMI = body mass index, TmP/GFR = tubular maximum phosphate reabsorption per glomerular filtration rate, XLH = X-linked hypophosphatemia

#### Laboratory test trends over 48 weeks of burosumab treatment

**Fig. 2:**
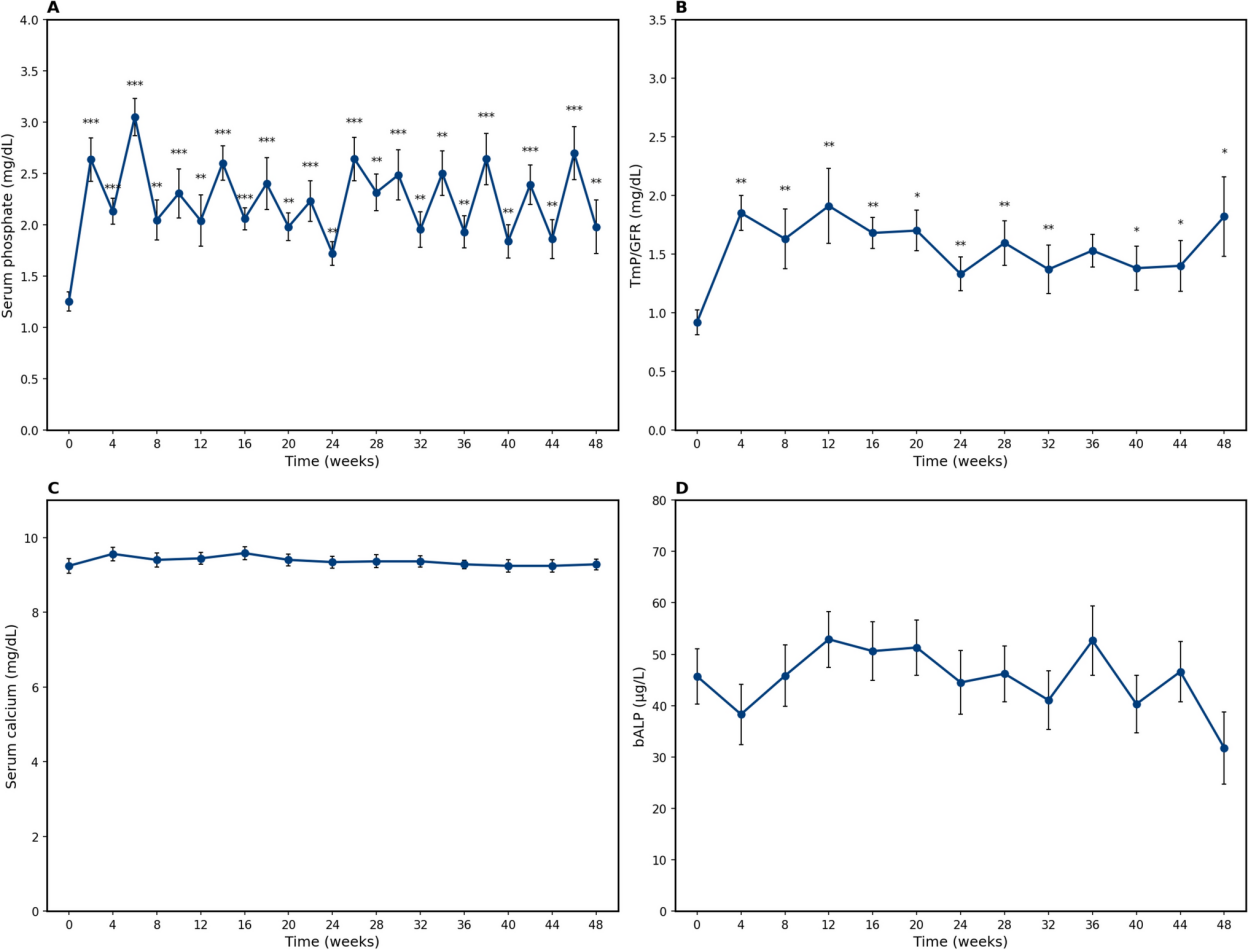
Effect of burosumab on serum phosphate (**A**), TmP/GFR (**B**), serum calcium (**C**) and bALP (**D**) up to week 48. * represents a p-value of < 0.05, ** = p < 0.01 and *** = p < 0.001. bALP = bone alkaline phosphatase, TmP/GFR = tubular maximum phosphate reabsorption per glomerular filtration rate

Even in this analysis, after the first administration of burosumab, serum phosphate increased from a baseline of 1.3 mg/dL (IQR 1.0–1.5) to a maximum value of 2.6 mg/dL (IQR 2.4–2.9) at week 2 and decreased to 2.1 mg/dL (IQR 1.9–2.4) at week 4, remaining significantly higher than baseline throughout the dosing interval. Although showing a slight decreasing trend, median serum phosphate levels remained higher than baseline throughout the observation period, both at midpoints and endpoints of dosing intervals, with the observed value at week 48 being 2.0 mg/dL (IQR 1.7–2.7) (Fig. 2A).

Similarly, TmP/GFR increased from a baseline of 0.9 mg/dL (IQR 0.8–1.1) and reached higher values at each endpoint of the dosing interval, with the observed value at week 48 being 1.8 mg/dL (IQR 1.2–2.5) (Fig. 2B). No significant difference in the trend of serum calcium levels was observed between the endpoints of dosing intervals (Fig. 2C). Conversely, for bALP (Fig. 2D), there seemed to be an initial upward trend (median 45.7 µg/L [IQR 29.5–53.6 µg/L]) followed by a decline (median 31.8 µg/L IQR [20.9–38.7 µg/L]), although this was not statistically significant (p = 0.199). No difference was also observed between baseline PTH levels (median 29.5 ng/L [IQR 27.2–53.8 ng/L]) versus 48 weeks (median 37.3 ng/L [IQR 36.4–44.6 ng/L]), (p = 0.377).

#### Factors associated with serum phosphate at midpoints in subgroup analysis

Consistent with the analysis conducted in the entire cohort, the mean serum phosphate at midpoints during the first 24 weeks was also calculated for patients from the University Hospital of Padova. Four patients had mean serum phosphate values below 2.5 mg/dL, and seven had values equal to or greater than 2.5 mg/dL. In particular, there was a significant (p < 0.01) difference between the two groups with respective median values of 2.1 mg/dL (IQR 1.9–2.3) and 2.8 mg/dL (IQR 2.7–3.4). Different trends in serum phosphate in the two groups at the midpoints and endpoints of the dosing interval up to week 48 are shown in Supplementary Fig. 2.

The two groups—lower (< 2.5 mg/dL) and higher (≥ 2.5 mg/dL) mean serum phosphate at 24-week midpoints—did not significantly differ in terms of age, sex, anthropometric parameters, serum phosphate, TmP/GFR, serum and urinary calcium, creatinine and 25-OH-vitamin D (p > 0.05 for all comparisons). However, both PTH (63 ng/L, IQR 34–85 vs. 29 ng/L, IQR 24–38, p = 0.04) and iFGF23 (240 pg/mL, IQR 144–399 vs. 103 pg/mL, IQR 67–161, p = 0.04) were higher in subjects with low mean serum phosphate at midpoints. A univariable regression analysis was performed using mean serum phosphate at 24-week midpoints as the dependent variable. These results revealed significant associations for both PTH (b = − 0.014, p = 0.043) and iFGF23 (b = − 0.0026, p = 0.035), indicating that higher levels of these variables are predictive of lower mean serum phosphate at midpoints. The small sample size precluded the possibility to perform multivariable regression analysis.

We next performed ROC curve analysis to assess the discriminative power of PTH and iFGF23 in identifying patients with lower (< 2.5 mg/dL) versus higher (≥ 2.5 mg/dL) mean serum phosphate at 24-week midpoint. The AUC for PTH is 0.79, while for iFGF23 it is 0.88. The optimal cutoffs were determined to be 43.1 ng/L (reference range: 6.5–36.8 ng/L) for PTH and 209 pg/mL (reference range: 23.2–95.4 pg/mL) for iFGF23.

Finally, in this subgroup we also calculated the mean serum phosphate levels at midpoints over 48 weeks. Only one patient, who was categorized in the higher (≥ 2.5 mg/dL) group at 24 weeks, transitioned to the lower (< 2.5 mg/dL) group at 48 weeks, while all other patients remained in their original categories.

#### Patient-reported outcome trends during treatment with burosumab in subgroup analysis

**Fig. 3:**
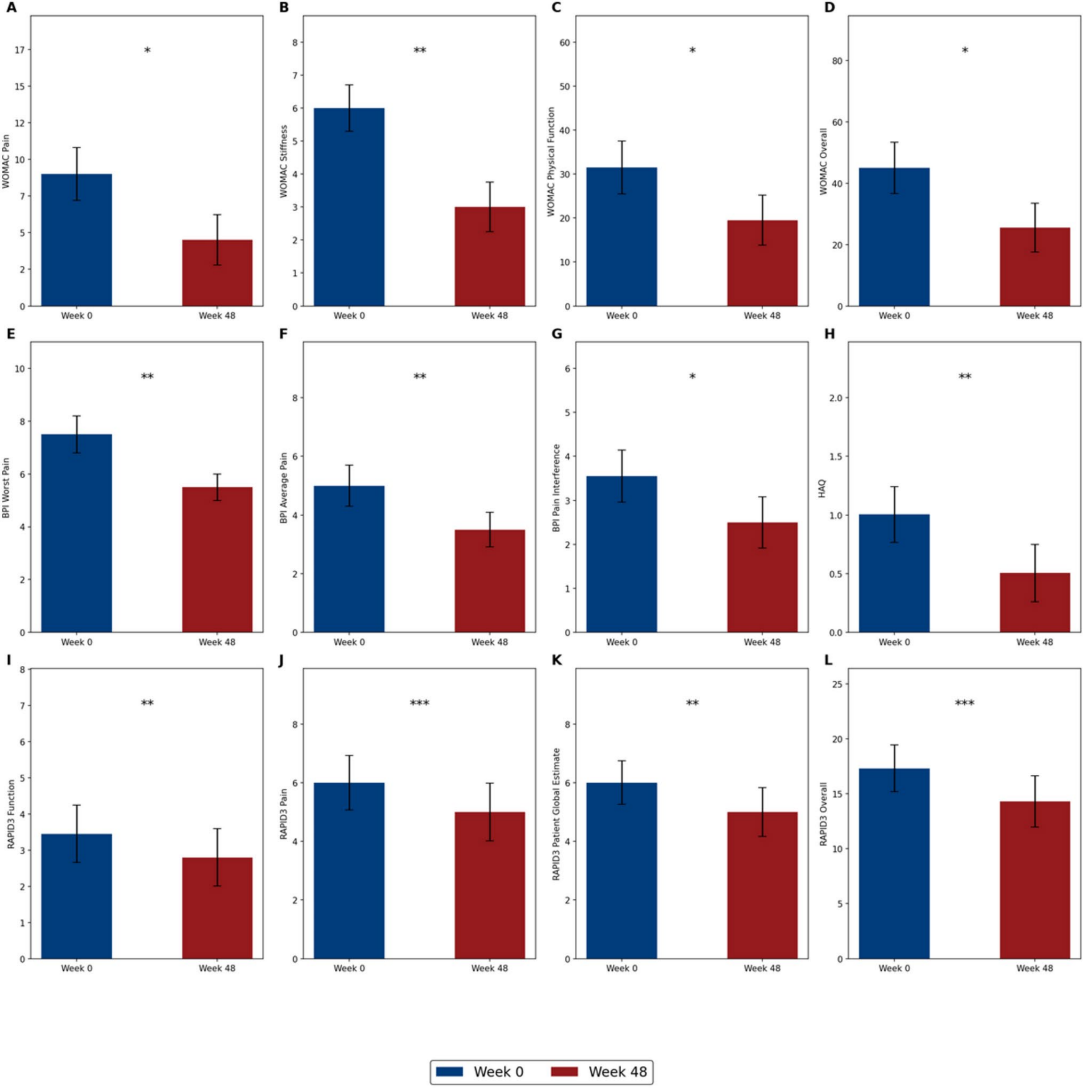
Patient-reported outcome scores after burosumab treatment are shown at baseline and at week 48 for the following measures: WOMAC Pain (**A**), WOMAC Stiffness (**B**), WOMAC Physical Function (**C**), WOMAC Overall (**D**), BPI Worst Pain (**E**), BPI Average Pain (**F**), BPI Pain Interference (**G**), HAQ (**H**), RAPID3 Function (**I**), RAPID3 Pain (**J**), RAPID3 Patient Global Estimate (**K**), and RAPID3 Overall (**L**). BPI = The Brief Pain Inventory, HAQ = Health Assessment Questionnaire, RAPID3 = Routine Assessment of Patient Index Data 3, WOMAC = Western Ontario and McMaster Universities Osteoarthritis Index. * represents a p-value of < 0.05, ** = p < 0.01 and *** = p < 0.001

Treatment with burosumab significantly improved patient-reported outcomes at week 48 compared to baseline. These outcomes were collected from 8 patients out of 11 from Padova. Regarding the WOMAC scale, there was an improvement in Pain, Stiffness, Physical Function, and Overall scores (Fig. 3A–D). Specifically, the Overall score improved from a median of 45.0 (IQR 31.1–72.2) at week 0 to a median of 25.5 (IQR 21.4–51.0) at week 48, with a median percentage improvement of 20.0% (IQR 10.5–45.4, p = 0.03). For the WOMAC Pain score, the median improved from 9.0 (IQR 6.4–13.6) at week 0 to 4.5 (IQR 3.0–9.1) at week 48, with a median percentage improvement of 39.3% (IQR 16.7–61.5, p = 0.02).

Similarly, the WOMAC Stiffness and Physical Function scores showed significant improvements with median percentage changes of 29.1% (IQR 20.2–59.7, p < 0.01) and 17.3% (IQR 7.1–37.7, p = 0.04), respectively. Additionally, significant reductions were observed in BPI Worst Pain, BPI Average Pain, and BPI Pain Interference scores (Fig. 3E–G). For BPI Worst Pain, the median score improved from 7.5 (IQR 5.8–8.6) at week 0 to 5.5 (IQR 4.4–6.0) at week 48, with a median percentage improvement of 29.1% (IQR 18.8–47.0, p < 0.01).

BPI Average Pain and BPI Pain Interference scores also showed notable improvements with median percentage changes of 26.8% (IQR 20.9–50.0, p < 0.01) and 11.8% (IQR 6.3–41.0, p = 0.04), respectively. HAQ and RAPID3 scores (Fig. 3H–L) also showed significant improvements. The HAQ score improved from a median of 1.0 (IQR 0.4–1.4) at week 0 to 0.5 (IQR 0.2–1.2) at week 48, with a median percentage improvement of 37.0% (IQR 13.3–52.5, p < 0.01). RAPID3 Function, Pain, Patient Global Estimate, and Overall scores demonstrated similar trends, with significant reductions and median percentage improvements of 19.5% (IQR 8.7–36.8, p < 0.01), 16.7% (IQR 11.7–37.5, p < 0.001), 16.7% (IQR 13.2–36.1, p < 0.01), and 16.0% (IQR 11.6–38.1, p < 0.001), respectively. Age was not correlated with any of the baseline scores nor with the extent of variation at 48 weeks (p > 0.05 for all comparisons). Similarly, no significant differences between the two sexes were found in baseline scores and the extent of variation at 48 weeks (p > 0.05 for all comparisons).

Regarding baseline laboratory tests, neither serum phosphate, TmP/GFR, PTH, nor FGF23 levels were correlated with the baseline score values; similarly, serum phosphate at week 48 was not correlated with the extent of score variation (p > 0.05 for all comparisons).

On the contrary, the extent of the change in scores for some measures was positively correlated with the increase in serum phosphate at week 48 compared to baseline, specifically WOMAC Pain (r = 0.94, p = 0.02), WOMAC Physical Function (r = 0.89, p = 0.03), WOMAC Overall (r = 0.94, p = 0.02), BPI Worst Pain (r = 0.98, p < 0.001), and BPI Average Pain (r = 0.88, p = 0.02).

## Discussion

The main findings from this real-world study show that in patients with confirmed XLH, burosumab improved serum phosphate levels and clinical outcomes, with baseline phosphate, PTH, and FGF23 levels predicting response, helping to guide personalized treatment.

XLH presents a complex challenge in the field of metabolic bone disorders, and burosumab has emerged as a revolutionary treatment. The efficacy of burosumab in increasing serum phosphate concentrations has been documented in several studies [15, 17, 25–28], as well as through various meta-analyses and systematic reviews [29, 30].

Real-world Italian data from our study align with these findings. In fact, we observed an improvement in mean serum phosphate at midpoints during 24 weeks of treatment, showing an 86% increase compared to baseline phosphate levels before starting burosumab therapy. Notably, serum phosphate levels remained consistently higher than baseline at all timepoints analyzed. The efficacy of burosumab on serum phosphate levels remained evident even in those of our patients whose observation period was extended from 24 to 48 weeks. In these 11 patients, PTH levels did not show any significant changes compared to baseline at any time point up to 48 weeks (p > 0.05 for all comparisons). FGF23 measurements were not available for these patients, primarily because, during treatment with burosumab, analytical interferences are recognized to limit the ability to interpret FGF23 assays [31]. In these patients we also observed a concomitant increase in TmP/GFR, further highlighting the drug’s impact on the underlying pathophysiological mechanisms of the disease. In addition to the biochemical improvement, our findings are consistent with the literature, showing that patient-reported outcomes, including pain, stiffness, physical function, and QoL scores, improved from baseline to week 48 [17, 27, 32–34].

However, when comparing our data with findings from the pivotal trial [17], we observed a somewhat less pronounced increase in serum phosphate levels. Specifically, 63% of patients (17 out of 27) achieved normal mean serum phosphate levels at the 24-week midpoints in the entire study population, while 55% (6 out of 11) reached this target at the 48-week midpoints. In our view, this may be explained by differences in the clinical characteristics of adult XLH patients eligible for burosumab access in Italy, initially through the EAP and then via reimbursement by the National Health System. As already mentioned, these access criteria included the presence of active fractures/pseudofractures, moderate to severe pain impacting QoL, and/or the need for orthopedic, dental or spinal surgery for a predefined period before and after the intervention. Alignment with these criteria may have prioritized individuals with a more severe disease profile compared to participants included in the randomized controlled trials conducted on burosumab use. This is supported by the observation of lower baseline serum phosphate and TmP/GFR levels than the pivotal trial (2.0 ± 0.3 mg/dL and 1.7 ± 0.4 mg/dL, respectively) [17], despite all patients being on conventional therapy. Our results are not surprising because, when trying to identify potential baseline parameters that could serve as predictors of serum phosphate increase, we found that lower serum phosphate levels were associated with a less pronounced increase in this ion during burosumab therapy. Furthermore, our results also demonstrated that the biochemical response to burosumab was negatively influenced by baseline values of PTH and FGF23. Even if it is known that serum PTH levels tend to be elevated (54.4–100%), and FGF23 levels are abnormally high (74.2–100%) in most patients with XLH [1, 19, 28], the correlation between these two factors and the response to burosumab treatment has not been extensively reported in previous studies. In the present study, patients achieving a mean serum phosphate levels at midpoints of less than 2.5 mg/dl had baseline values of PTH and FGF23 approximately two-fold higher compared to subjects with a better biochemical response to the drug. In our opinion, these data could support the hypothesis that PTH and, based on the multivariate regression analysis, especially FGF23 levels may modulate the effect of burosumab on phosphate homeostasis in XLH patients.

It has been reported that the majority of adult XLH patients experience a reduced QoL due to pain, physical disability, abnormal gait, and global fatigue [35]. The effectiveness of burosumab in improving patient-reported outcomes has been documented in previous studies [17, 27, 32–34]. For example, in a large international Phase 3 RCT involving 134 adults with XLH, the burosumab group showed significant improvements in patient-reported outcomes and healing of fractures and pseudofractures at 24 weeks [17]. Subsequent study extensions demonstrated that these improvements continued beyond 24 weeks, particularly in terms of pain, stiffness, fatigue, and functional capacity [25]. The findings of this study are consistent with the literature, showing that patient-reported outcomes, including pain, stiffness, physical function, and QoL scores, improved from baseline to week 48.

Interestingly, all patients who completed the patient-reported outcome questionnaires at 48 weeks showed an improvement in their scores compared to baseline, regardless of whether the serum phosphate target was reached. However, it should also be noted that the extent of the change in scores for WOMAC Pain, WOMAC Physical Function, WOMAC Overall, BPI Worst Pain, and BPI Average Pain were positively correlated with the increase in serum phosphate at week 48 compared to baseline. This suggests that while clinical improvement can occur independently of reaching the phosphate target, a stronger biochemical response may enhance the extent of clinical benefit.

### Study limitations

While our study offers valuable insights into the predictors of response to burosumab in patients with XLH, several limitations should be considered. First, the sample size was relatively small and this is largely attributable to the rarity of XLH, which inherently restricts patient recruitment in clinical research. The small sample size, particularly in subgroup analyses, may limit the generalizability of our findings. The limited number of participants also restricted our ability to conduct multivariate regression analyses to fully explore the interactions between different baseline variables and treatment response. However, the fact that XLH is a rare disease had to be taken into account. Moreover, the specific criteria for access to the drug in Italy may have selected a population different from those described in the literature. However, we think that exploring the effects of burosumab use in adult XLH patients in a real-world setting may be of interest.

In addition, XLH, is a lifelong condition and the observational period of 24–48 weeks might not be sufficient to capture the long-term effectiveness and safety of burosumab, highlighting the need for extended studies. Furthermore, our study did not include a control group, making it difficult to attribute improvements in patient-reported outcomes solely to burosumab without considering other potential confounding factors such as changes in diet, physical activity, or concomitant treatments.

## Conclusion

In summary, our findings highlight the critical importance of baseline serum phosphate, PTH, and FGF23 levels as key predictors of the response to burosumab treatment in patients with XLH. Additional research should focus on optimizing treatment protocols and identifying other potential predictors of response to burosumab. By advancing our understanding of these dynamics, future studies can contribute to more effective, individualized treatment strategies and improved QoL for patients with X-linked hypophosphatemia.

## Supplementary Information

Below is the link to the electronic supplementary material.

Supplementary file1 (TIFF 20493 KB)

Supplementary file2 (TIFF 8397 KB)

Supplementary file3 (DOCX 12 KB)

## Data Availability

Raw data can be obtained upon motivated request from the corresponding author.
