# Supplementary figures and images for: Predictors of response to burosumab in adults with X-linked hypophosphatemia: real-world data from an Italian cohort
Source: J Endocrinol Invest. 2025 May 5;48(8):1857–69. doi: 10.1007/s40618-025-02596-3 (PMC12313718; doi:10.1007/s40618-025-02596-3)

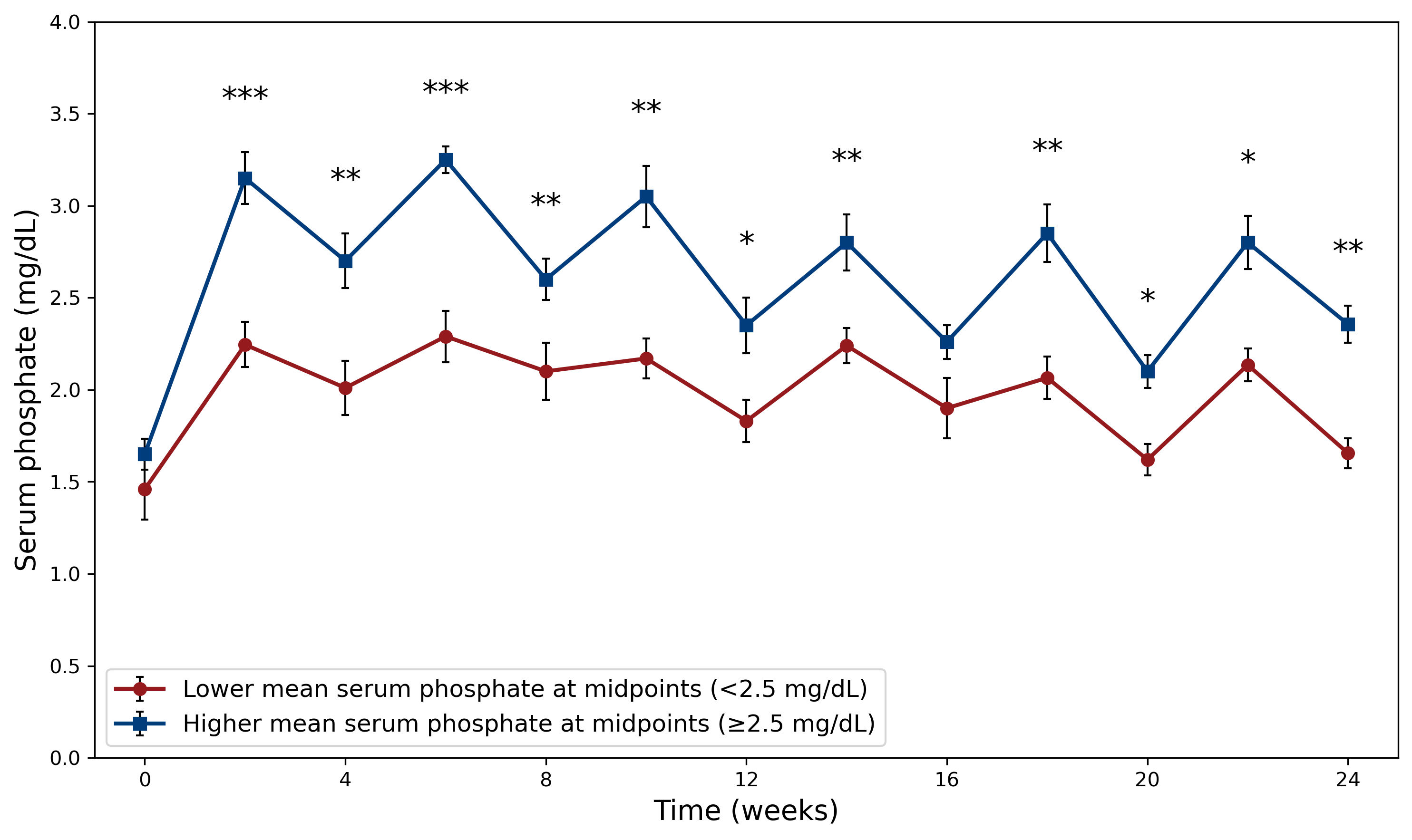

Supplement: Supplementary file 1 — Supplementary file1 (TIFF 20493 KB) [file 40618_2025_2596_MOESM1_ESM.tiff]

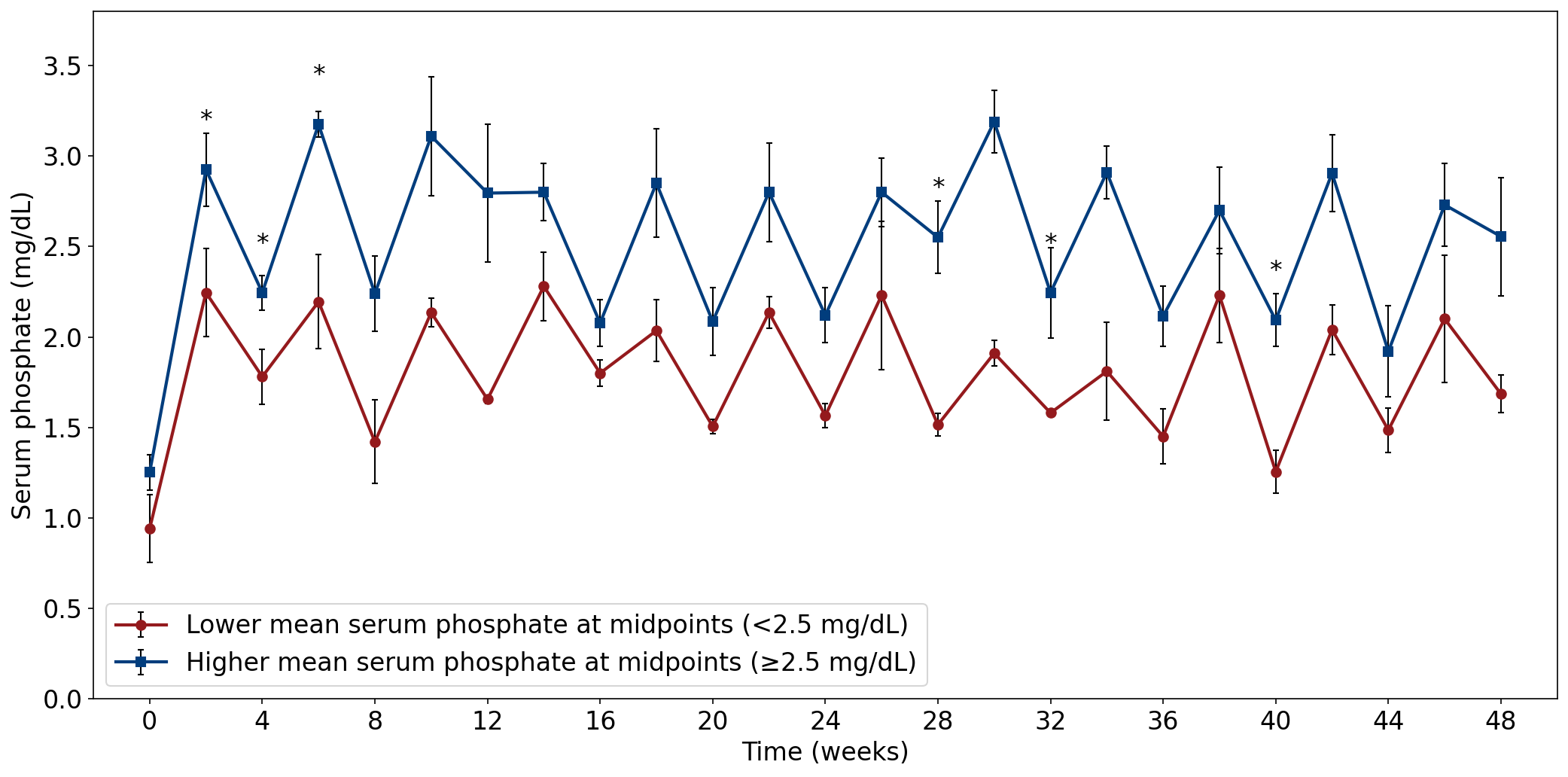

Supplement: Supplementary file 2 — Supplementary file2 (TIFF 8397 KB) [file 40618_2025_2596_MOESM2_ESM.tiff]
