# Supplementary material for: Predictors of response to burosumab in adults with X-linked hypophosphatemia: real-world data from an Italian cohort
Source: J Endocrinol Invest. 2025 May 5;48(8):1857–69. doi: 10.1007/s40618-025-02596-3 (PMC12313718; doi:10.1007/s40618-025-02596-3)
Supplement: Supplementary file 3 — Supplementary file3 (DOCX 12 KB) [file 40618_2025_2596_MOESM3_ESM.docx]

**Figure legend**

**Supplementary Material Figure 1.** Trends of serum phosphate up to week 24 and 48 weeks. A, Trends of serum phosphate up to week 24 comparing patients with lower and higher mean serum phosphate at midpoints. * represents a p-value of <0.05.

**Supplementary Material Figure 2.** Trends in serum phosphate up to week 48 in patients with lower and higher mean serum phosphate at midpoints. * represents a p-value of <0.05.
